# Monolithic DSSC/CIGS tandem solar cell fabricated by a solution process

**DOI:** 10.1038/srep08970

**Published:** 2015-03-11

**Authors:** Sung Hwan Moon, Se Jin Park, Sang Hoon Kim, Min Woo Lee, Jisu Han, Jin Young Kim, Honggon Kim, Yun Jeong Hwang, Doh-Kwon Lee, Byoung Koun Min

**Affiliations:** 1 Clean Energy Research Center, Korea Institute of Science and Technology, Hwarang-ro 14-gil 5, Seongbuk-gu, Seoul. 136–791, Republic of Korea; 2 Department of Chemical and Biological Engineering, Korea University, 145, Anam-ro, Seongbuk-gu, Seoul 136–713, Republic of Korea; 3 Center for Materials Architecturing, Korea Institute of Science and Technology, Hwarang-ro 14-gil 5, Seongbuk-gu, Seoul, 136–791, Republic of Korea; 4 Photo-electronic Hybrids Research Center, Korea Institute of Science and Technology, Hwarang-ro 14-gil 5, Seongbuk-gu, Seoul. 136–791, Republic of Korea; 5 Green School, Korea University, 145, Anam-ro, Seongbuk-gu, Seoul 136–713, Republic of Korea

## Abstract

Tandem architecture between organic (dye-sensitized solar cell, DSSC) and inorganic (CuInGaSe_2_ thin film solar cell, CIGS) single-junction solar cells was constructed particularly based on a solution process. Arc-plasma deposition was employed for the Pt interfacial layer to minimize the damage to the layers of the CIGS bottom cell. Solar cell efficiency of 13% was achieved, which is significant progress from individual single-junction solar cells (e.g., 7.25 and 6.2% for DSSC and CIGS, respectively).

Tandem architecture of solar cells, wherein two or more sub-cells with complementary absorption characteristics are stacked and connected either in series or in parallel, is one of the exciting approaches to increase the efficiency effectively of solar cells beyond the Shockley-Queisser limit of single-junction devices^1^,^2^,^3^. For example, dual- and triple-junction solar cells based on III–V compound semiconductor materials such as InGaP/GaAs/InGaAs have been demonstrated to show over 37% power conversion efficiency (PCE)^4^. Moreover, several other photovoltaic materials, including a-Si, CIGS, and various polymers, have also been explored in order to fabricate efficient tandem solar cells^5^,^6^,^7^,^8^.

In addition to the tandem structure achieved with similar class materials, two substantially different solar cell materials have also been incorporated into tandem solar cell construction. For example, tandem solar cells fabricated with a-Si and a polymer have been demonstrated to show enhanced voltage and power conversion efficiency compared to conventional single-junction solar cells^9^,^10^,^11^. Furthermore, the tandem architecture of a DSSC with GaAs or polymer is reported to show high voltages of 1.8 and 1.36 V, respectively^12^,^13^. Recently, Liska et al. and Wenger et al. suggested the possibility of a tandem type of architecture with DSSC and CIGS based on mechanical stacking as well as a monolithic conjunction^14^,^15^. Such a tandem construction of DSSC with CIGS seems to be an ideal design owing to the optical band-gaps of DSSC (~1.7 eV) and CIGS (~1.1 eV), making them suitable for use as top and bottom cells, respectively (see Fig. 1). Furthermore, CIGS offers the feasibility of tuning the band-gap by adept control of its composition. This property is particularly beneficial for tandem solar cell applications. For example, by substituting In with Ga and/or Se with S, the band-gap of CIGS can be gradually increased in the range between 1.0 (CuInSe_2_) and 2.4 eV (CuGaS_2_)^16^. In addition, from the viewpoint of manufacturing costs, the DSSC/CIGS tandem solar cell would be a competitive option, given the advantage of the economic viability of DSSCs prepared by solution processes. However, to realize a further reduction in the cost of DSSC/CIGS tandem solar cells, it is necessary to develop low-cost and high-throughput solution processing methods (e.g., printing) for the fabrication of the bottom CIGS cell, which is currently fabricated by a vacuum-based method such as co-evaporation. Recently, solution-based fabrication methods for CIGS thin films have attracted much attention due to their potential for realizing low-cost and printable solar cells^17^. The highest efficiencies of solution processed CIGS thin film solar cells have been reported to be 15.2% (hydrazine based)^18^ and 12% (non-hydrazine based)^19^. Among the various solution-based synthesis methods, the fabrication of CIGS thin films using nanoparticle ink has potential. This method offers the advantage of fabricating CIGS thin films with uniform and well-controlled compositions by starting from stoichiometry-controlled CIGS nanoparticles, thereby eliminating the impurity phases that could significantly deteriorate the device performance^20^. With subsequent sintering of the as-prepared film, the nanoparticles are converted to uniform films of high crystallinity and a large grain size, which in turn lead to a high solar-to-electricity conversion efficiency of 12%, as demonstrated by Guo et al.^19^

One of the major issues in the fabrication of efficient tandem DSSC/CIGS cells (Fig. 1) is to control the electrical/optical properties of the interface between the DSSC and the CIGS. A Pt-based catalytic layer is deposited on the transparent conducting oxide (TCO) layer of the CIGS thin film solar cell such as Al-doped ZnO (AZO) and Sn-doped In_2_O_3_ (ITO)^21^. The role of the Pt catalytic layer is effectively to reduce the overpotential for the reduction of the I_3^-^_ ions to I^-^ ions by the photoelectrons coming out from the underneath CIGS solar cell. However, the existence of the Pt catalytic layer inevitably decreases the optical transmittance, which should lead to a decrease in the photocurrent from the bottom cell and to a photocurrent mismatch in the tandem solar cell. Therefore, it is desirable to prepare Pt catalysts with high catalytic properties and high optical transmittance simultaneously. The Pt catalytic layer in a DSSC is conventionally prepared via a simple thermal decomposition method using a Pt precursor (e.g., H_2_PtCl_6_), followed by sintering at 380–450°C under ambient conditions^22^. However, this method cannot be applied during the fabrication of a DSSC/CIGS tandem cell, as the bottom CIGS cell, fabricated prior to the Pt layer, is vulnerable to high-temperature treatments. Therefore, it is crucial to identify a suitable low-temperature preparation method for the fabrication of the Pt layer.

In this study, a Pt interfacial layer was deposited by the arc-plasma deposition (APD) technique, which is considered to be a low-temperature and softer deposition method that could minimize the damage to the AZO and CIGS absorber films. The DSSC/CIGS tandem solar cell was fabricated via a simple solution-based process by preparing the CIGS absorber film for the bottom cell using CIGS nanoparticle ink. We have therefrom demonstrated a DSSC/CIGS tandem solar cell that shows a higher solar cell performance (PCE of 13.0%) than the corresponding single-junction devices of DSSC (~7.25%) or CIGS (~6.2%). Note that this value is higher than the champion cell efficiencies of the constituent single-junction solar cells in the literature, i.e., 11.2% of N719 dye-based DSSC^23^ and 12% of nanoparticle ink-based CIGS^19^.

## Results

**Figure 1:**
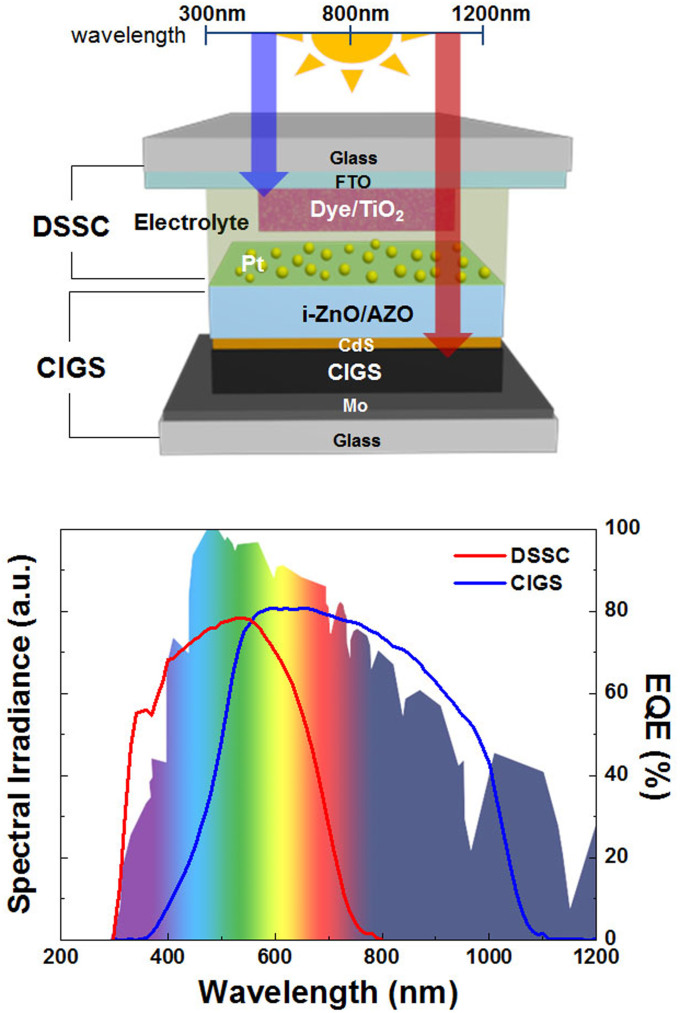
Schematic of the DSSC/CIGS tandem solar cell structure (upper), and spectral irradiance of solar light adapted from reference 3 and external quantum efficiencies of a DSSC (red) and a CIGS (blue) single-junction solar cell used in the study (bottom).

**Figure 2:**
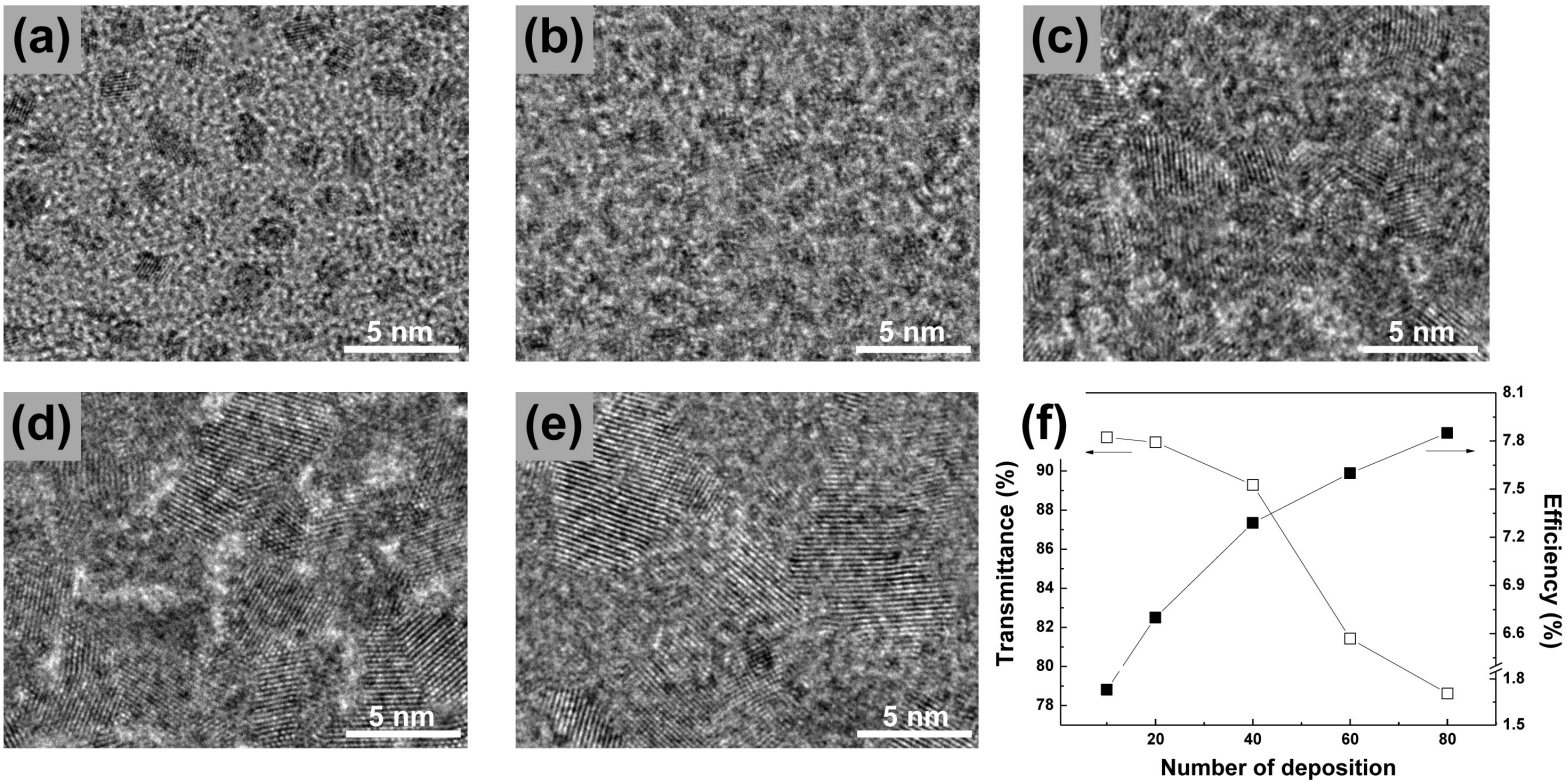
Transmission electron microscopy (TEM) images of Pt nanoparticles deposited on amorphous carbon films by the arc-plasma deposition (APD) method with different plasma pulses (a) 10, (b) 20, (c) 40, (d) 60, and (e) 80, and optical transmittance at 800 nm of APD-Pt layers on glass substrates (left y-axis in (f)) and conversion efficiencies of DSSCs with APD-Pt catalyst (right y-axis in (f)) as a function of the plasma pulses.

As mentioned earlier, one of the most important components in the DSSC/CIGS tandem architecture is the Pt catalyst deposited on the AZO layer of a CIGS thin film solar cell. The APD method^24^,^25^ involves the direct evaporation of a solid metal target into a highly ionized plasma pulse in vacuum (~3x10^−5^ Torr). The kinetic energy of the evaporated metal nanoparticles is relatively low (tens of eV), and hence these nanoparticles can be deposited onto a substrate without causing significant damage to the substrate surface. The pulsed deposition mode (i.e., enough idle time between plasma pulses) along with the low kinetic energy of the evaporated nanoparticles also prevents the temperature from increasing in the samples (<100°C). Another advantage of the APD method is that the amount of metal deposits can be controlled easily and accurately by changing deposition parameters such as the number of plasma pulses (*n*), the discharge condenser voltage (*V*), and the discharge condenser capacitance (*C*). Figs. 2a–e show transmission electron microscopy (TEM) images of the Pt catalysts deposited using the APD method with different values of *n* (10, 20, 40, 60, and 80), where glass substrates with an amorphous carbon film were used for the coverage estimation. Isolated Pt nanoparticles with a diameter of ~2 nm were observed after 10 plasma pulses (Fig. 2a), and the density of Pt nanoparticles increases with an increase in the number of plasma pulses, resulting in an almost continuous Pt film after 60 pulses. The catalytic properties of the APD-deposited Pt layer was investigated in terms of the conversion efficiency of DSSCs incorporating APD-Pt catalysts with different values of *n* (Fig. 2f, filled squares), where the conversion efficiency increases with an increase in the number of plasma pulses. This result shows that more Pt catalysts with more plasma pulses would be beneficial to enhance the device performance of the single-junction DSSC. In the case of a DSSC/CIGS tandem solar cell, however, the optical transmittance of the catalytic layer is also very important, because it directly influences light harvesting by the bottom CIGS solar cell, and the resulting photocurrent generation. In this context, the ultraviolet-visible (UV-Vis) spectra of Pt layers deposited on glass substrates using the APD method with different values of *n* have been investigated. Given that the bottom CIGS cell can only absorb photons that have passed through the top DSSC, transmittance values at 800 nm are compared (see Fig. 1). As shown in Fig. 2f (open squares), the optical transmittance at 800 nm decreases with an increase in the number of plasma pulses, especially after 40 pulses, which is consistent with the TEM analysis. Owing to the trade-off between the DSSC performance and light harvesting by the bottom CIGS, the optimal condition for the Pt deposition by APD was set to 40 pulses. It should be noted that the conversion efficiency of a DSSC with a Pt layer deposited by APD (40 pulses, 7.25 (±0.26)%) was slightly higher than that of a DSSC with Pt layer deposited by the conventional thermal sintering method (7.16%, see Supplementary Information, Fig. S1).

In order to realize the solution processed fabrication of a DSSC/CIGS tandem solar cell, we adopted the nanoparticle ink-based coating method for the deposition of CIGS absorber film. In this study, the method that was originally developed by Guo et al. was slightly modified for the fabrication of the absorber film of the CIGS bottom cell (see Methods section). Briefly, the CIGS film was formulated from CIGS nanoparticles, which were synthesized via the hot injection route using Cu, In, and Ga acetylacetonate precursors and elemental S. Subsequently, ink composed of CIGS nanoparticles and hexanethiol was doctor-bladed and preheated under ambient conditions. After a sodium treatment, the film was annealed under selenium vapor to allow the substitution of Se for S and to facilitate crystal growth^19^.

**Figure 3:**
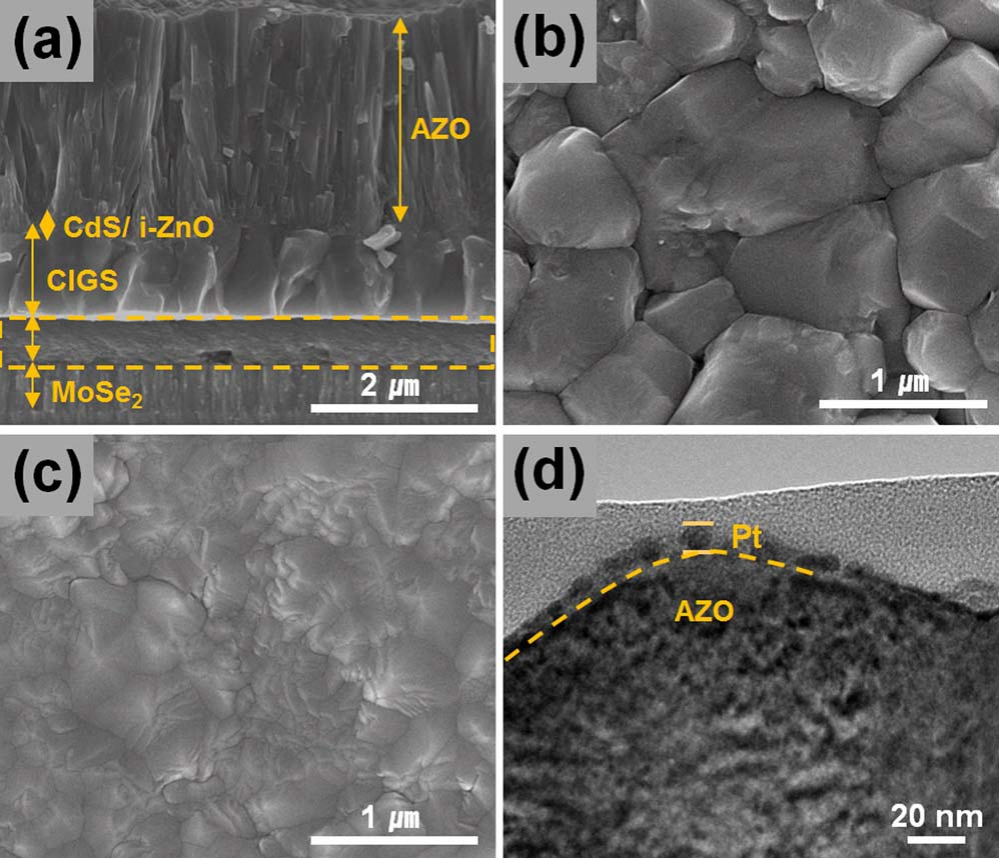
Cross-sectional scanning electron microscope (SEM) image of bottom CIGS cell (AZO/i-ZnO/CdS/CIGS/Mo) (a), top-view SEM images of the CIGS film only (b) and AZO/i-ZnO/CdS/CIGS film (c), and TEM image of Pt nanoparticles deposited on AZO/i-ZnO/CdS/CIGS film by APD.

**Figure 4:**
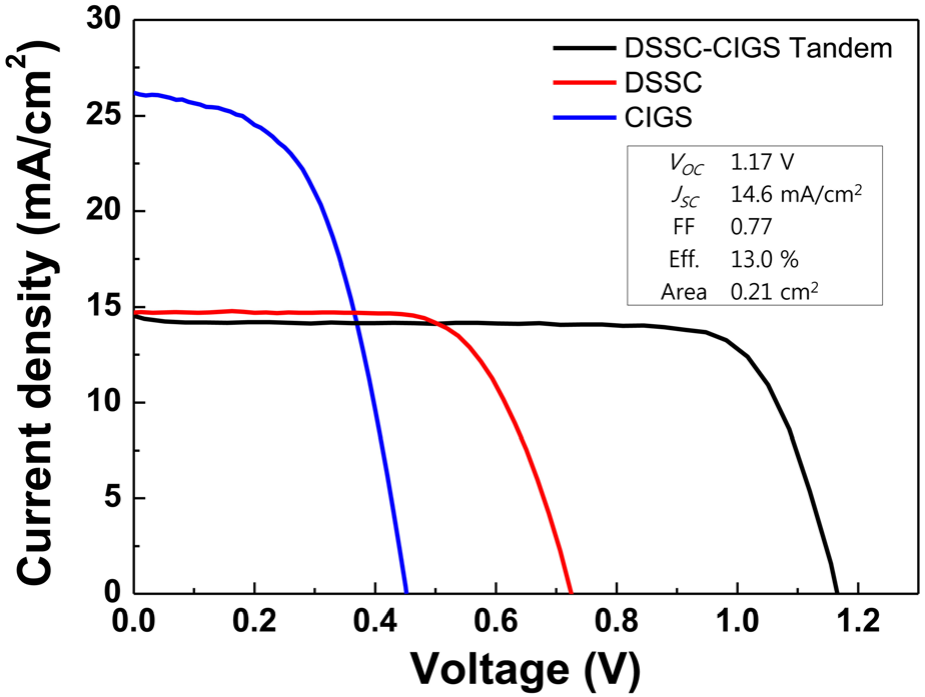
Current density-voltage (*J–V*) characteristics of DSSC/CIGS tandem solar cell and DSSC and CIGS single-junction solar cells under 1 sun illumination.

Fig. 3a shows the cross-sectional configuration of the CIGS bottom cell. As shown in the figure, the CIGS thin film shows large-sized grains (~1 μm) with a very low degree of porosity (Figs. 3a and b). Moreover, we could observe an amorphous layer with a thickness of 540 nm at the interfacial region of the Mo substrate, as marked by the dashed lines in Fig. 3a. This amorphous layer could be a residual carbon layer of the type often observed in solution-processed CIGS thin films^26^,^27^. This hypothesis was further substantiated by Auger electron spectroscopy (AES) for an elemental analysis, which confirmed the presence of carbon near the Mo layer (Supplementary Information, Fig. S2). In addition, the selenization of the Mo substrate resulted in the formation of a MoSe_2_ layer with a thickness of 580 nm. Furthermore, as shown in Fig. 3a, a CdS buffer layer and i-ZnO and AZO window layers were deposited onto the top surface of the CIGS absorber film. Owing to the deposition of the AZO film, the top surface of the CIGS bottom cell appears to be very dense (Fig. 3c). In particular, we deposited a thicker AZO film (2500 nm), approximately five times thicker than the conventional CIGS solar cell (500 nm), in order to protect the underneath CdS/CIGS layers during the deposition of Pt. Pt deposition by APD (40 pulses) resulted in the formation of smaller Pt nanoparticles on the top of the AZO surface, corresponding to the single-layer thickness of Pt particles (Fig. 3d). The representative single-junction CIGS thin film solar cell (closest one to the average efficiency value, 6.1 ± 0.5%, among the five cells) formed using the ZnO/CdS/CIGS films deposited onto Mo-coated glass shows a power conversion efficiency of 6.2%, with *J*_SC_, *V*_OC_, and FF values of 26.3 mA/cm^2^, 450 mV, and 52.3%, respectively (see Fig. 4).

The DSSC top cell was fabricated according to the conventional procedures reported in the literature^28^. As in the typical process, a mesoporous TiO_2_ film of thickness 20 μm was prepared by a paste-coating method, followed by sintering at 500°C. A TiO_2_ blocking layer was also deposited between the FTO and mesoporous TiO_2_ film in order to enhance the transmission of light^29^. N719 dye and I_3^-^_/I^-^ ions were employed as a sensitizer and redox couples, respectively. As mentioned earlier (Fig. 2), the efficiency of a DSSC varies with respect to the deposited amount of the Pt layer. For tandem cell fabrication, we used a DSSC top cell with an average efficiency of ~7.25%, as shown in Fig. 4.

Fig. 4 shows the *J*–*V* characteristics of the DSSC/CIGS tandem solar cell along with the DSSC and CIGS single-junction solar cells. The as-fabricated DSSC/CIGS tandem cell showed a power conversion efficiency of 13.0% (champion device) with an open-circuit voltage (*V*_OC_) of 1.17 V, a short-circuit current density (*J*_SC_) of 14.6 mA/cm^2^, and a fill factor (FF) of 0.77. The measured *V*_OC_ of the tandem cell (1.17 V) was in fairly good agreement with the sum of the individual average *V*_OC_ values for DSSC and CIGS single-junction cells (1.18 V). The plateau value of *J*_SC_ for the tandem device (14.2 mA/cm^2^) was slightly lower than that of the DSSC (14.7 mA/cm^2^). The *J*_SC_ of CIGS bottom cell in the tandem configuration cannot be measured with two terminal electrodes (on top of the DSSC and at the bottom of the CIGS). It is also far from simple to estimate it by covering the CIGS with a dummy DSSC cell, due to the difficulties in applying DSSC components identical to those used for the top cell in the tandem structure. Nevertheless, the observation that the *J*_SC_ of tandem cell is quite close to that of the DSSC suggests that the *J*_SC_ values from both sub-cells are well balanced.

The FF of the tandem cell (0.77) was found to be much higher than those of the constituent DSSC (0.68) and CIGS (0.52) samples. This can be explained in part by considering the series and shunt resistances (*R*_s_ and *R*_sh_) connected in series. In principle, the *R*_s_ of a series-connected tandem device should be the sum of the *R*_s_ values for DSSC and CIGS samples. The slope values, d*J*/d*V*, at *V*_OC_ of the tandem and single-junction devices (7.59 Ωcm^2^ for DSSC and 4.46 Ωcm^2^ for CIGS) were thus compared as approximations to *R*_s_ values. The measured d*J*/d*V* value at *V*_OC_ of the tandem device (7.41 Ωcm^2^) was found to be smaller than the sum of d*J*/d*V* values for the constituent single-junction cells, 12.05 Ωcm^2^. The discrepancy is likely due to the absence of the FTO contribution to *R*_s_ in the tandem configuration (Fig. 1). The *R*_s_ evaluation from the d*J*/d*V* value at high *V* regime of dark *J*–*V* curves (Fig. S3) also showed that the *R*_s_ of the tandem cell (1.1 Ωcm^2^) is smaller than the sum of the *R*_s_ values for DSSC (0.55 Ωcm^2^) and CIGS (1.2 Ωcm^2^). Similarly, it was proved that the series-connected shunt resistance (*R*_sh_ = (1.2 ± 0.5) × 10^4^ Ωcm^2^) is also determined by the higher value of the two sub-cells, i.e., *R*_sh_ of DSSC ((1.4 ± 0.8) × 10^4^ Ωcm^2^). In comparison, *R*_sh_ of CIGS was (3.1 ± 0.5) × 10^2^ Ωcm^2^. As a consequence, the slope of the tandem device in the range of 0 to 1.0 V looks as flat as a DSSC, in contrast to a CIGS cell. This feature is an additional advantage of a tandem cell, particularly when employing a single cell with high shunt conductance, such as a CIGS cell.

## Discussion

A hypothetical tandem cell with two subcells having exactly complementary absorption profiles that do not overlap at all would have the efficiency close to the sum of the individual cell efficiencies. It is clear that this is not the case with the present combination of DSSC and CIGS solar cells as there is some amount of overlap in the absorption profiles of DSSC and CIGS, as shown in Fig. 1. Although we could not measure how high the CIGS bottom cell performs in the tandem configuration, we could estimate each device parameter of the bottom cell as follows. The *V*_OC_ of CIGS bottom cell (operating in the tandem cell) is rather straightforward to estimate, that is, *V*_OC_(CIGS) = *V*_OC_(tandem) − *V*_OC_(DSSC) = 1.170 V − 0.725 V = 0.445 V. This value is slightly smaller than that of the single-junction CIGS cell (0.450 V), which is reasonable. Assuming that the subcell current densities are well matched, the *J*_SC_ of the CIGS bottom cell should be that of the tandem cell, i.e., 14.6 mA/cm^2^. Namely, the CIGS bottom cell loses its current by ~45% in comparison with the CIGS single cell (26.3 mA/cm^2^). If the FF of CIGS bottom cell improves to such an extent that compensates for the current loss, the bottom cell may operate at the efficiency of PCE = *V*_OC_ × *J*_SC_ × FF = (0.445 V)(14.6 mA/cm^2^)(0.79) = 5.1% having a *J*-*V* behavior shown in Fig. S4, where the FF of CIGS bottom cell, 0.79, was assumed to be slightly higher than that of tandem cell (0.77). The significant enhancement in FF value of CIGS bottom cell can be attributed to the reduction of shunt conductance that is in turn due to the series connection of shunting path of CIGS bottom cell to that of DSSC top cell as well as to the reduction of series resistance due to the absence of lateral collection path along the TCO layer. The sum of the resulting individual cell efficiencies is, thus, 7.3% (DSSC) + 5.1% (CIGS) = 12.4% (Tandem). If we used the highest efficiency value of our single junction DSSC (7.5%) the expected tandem cell efficiency can increase up to 12.6%. Although this estimation is very rough, we think it is enough to show that the measured tandem cell efficiency (13.0%) is not unrealistic. Besides this, the subcells used in tandem construction can be different from each single cell due to the insufficient reproducibility, as is implied by efficiency deviation (6.1 ± 0.5%) of single junction CIGS thin film solar cells.

It is worth noting that the present PCE (13.0%) is the highest value among the monolithic DSSC/CIGS tandem solar cells reported thus far^15^. This is significant progress in that the present device fabricated with solution-processed CIGS surpasses the DSSC/CIGS tandem devices based on the vacuum-processed CIGS^15^. However, we should also note that our current tandem solar cell devices are not stable enough mainly due to usage of corrosive idodine electrolyst (Fig. S5). The improvement of stability of the tandem cell device is under investigation in our lab by applying less corrosive electrolyte.

In brief, we present a strategy to construct an efficient DSSC/CIGS tandem architecture by solution-based coating method. A Pt-based interfacial catalytic layer is the most important component in a DSSC/CIGS tandem cell; therefore, its catalytic properties and optical transmittance should be well optimized to achieve a highly efficient tandem solar cell. The APD method was successfully employed for the Pt interfacial layer, showing best solar cell performance with 40 pulses. Notably, the DSSC/CIGS tandem solar cell revealed much higher solar cell performance (PCE of 13.0%) than the corresponding single-junction devices of DSSC (~7.25%) or CIGS (~6.2%) cells. Moreover, these are higher values than the champion cell efficiencies of the constituent single-junction solar cells, i.e., 11.2% from a N719 dye-based DSSC and 12% from a nanoparticle ink-based CIGS cell.

## Methods

### Fabrication of the DSSC cell

Fluorine-doped tin oxide (FTO) glasses (Pilkington, TEC-8) were washed in ethanol using an ultrasonic bath. To create a working electrode, the FTO layer was initially covered with 7.5 wt% Ti(IV) bis(ethyl acetoacetato)-diisopropoxide solution by a spin-coating method, after which TiO_2_ paste was deposited by doctor blade method. The paste was prepared using nanocrystalline TiO_2_ (25 nm in diameter) that was synthesized by a hydrothermal method^28^. The coated film was sintered at 500°C for 30 min in air. The sintered TiO_2_ film was dipped into a TiCl_4_ solution (0.04 M) at 70°C for 30 min and was resintered at 500°C for 30 min. The thickness of the prepared film is 20 μm, as measured by an Alpha-Step IQ surface profiler (KLA Tencor). After cooling down to 80°C, the film was dipped into a N719 dye solution (0.5 mM) at 40°C for 3 hr.

Pt-based catalytic layer as counter electrode was prepared by two methods: arc plasma deposition (APD) and thermal sintering. The APD method used a coaxial pulsed APD system (ULVAC, ARL-300) at room temperature under a 10^−5^ Torr vacuum to deposit platinum nanoparticles onto FTO glass, whereas the thermal sintering method was carried out by spreading a 7 mM solution of H_2_PtCl_6_ dissolved in 2-propanol onto FTO glass and followed by drying and annealing at 450°C for 15 min.

Two small holes were drilled into the counter electrode to introduce the electrolyte, and 60 μm thick surlyn (DuPont 1702) was used to assemble the finished counter electrode with the working electrode. The electrolyte was introduced into the assembled device through a hole on the counter electrode, where the electrolyte solution was composed of PMII (0.7 M), I_2_ (0.03 M), guanidine thiocyanate (0.05 M) and 4-tert-butylpyridine (0.5 M) in acetonitrile and valeronitrile (85:15 v/v).

### Preparation of the CIGS film and solar cell fabrication

For the preparation of CIGS film, CIGS nanoparticles were synthesized using the method proposed by Guo et al. 3 mmol of copper (II) acetylacetonate, 2.1 mmol of indium(III) acetylacetonate, 0.9 mmol of gallium(III) acetylacetonate and 15 ml oleylamine were mixed in a three-necked flask and heated to 130°C using a heating mantle under Ar. The solution was pumped under a vacuum at 130°C for 30 min for degassing and heated to 285°C under Ar. At 285°C, 9 ml of 1 M solution of elemental sulfur in oleylamine was swiftly injected in a three-necked flask under vigorous magnetic stirring and the reaction was kept at 285°C for 30 min before cooling down. After cooling down, the nanoparticles were precipitated with isopropyl alcohol (IPA) through centrifugation at 12000 RPM for 5 min. The supernatant was discarded and the precipitate was redispersed in hexane. This purification process was repeated several times to remove excess oleylamine. The precipitate was then dried using an evaporator under vacuum and redispersed in hexanethiol to form an ink with concentration of ~200 mg/ml.

To make a film, the ink was coated onto Mo-coated soda-lime glass by a doctor blade method under ambient conditions. After coating, the film was annealed on a hot plate preheated to 300°C for 1 min and the coating and annealing steps were repeated two times to obtain a suitable film thickness. Prior to selenization, the nanocrystal films were soaked in a 1 M NaCl solution for 10 min followed by selenization with elemental Se under Ar at 500°C for 20 min in a quartz tube.

A solar cell device was fabricated with the conventional Mo/CIGS/CdS/i-ZnO/AZO/Ni/Al structure and the case of a tandem solar cell was excepted Ni and Al electrodes. A 60-nm-thick CdS buffer layer was prepared on 0.5 M aqueous potassium cyanide (KCN) solution-treated CIGS film by chemical bath deposition (CBD), and i-ZnO (50 nm)/Al-doped ZnO (2500 nm) were deposited by the radio-frequency magnetron-sputtering method. A Ni (50 nm) and Al (500 nm) grid were prepared as a current collector by thermal evaporation. The active area of the completed single cell was 0.44 cm^2^.

### Fabrication of DSSC/CIGS tandem device

Tandem device was fabricated by assembling a working electrode (FTO/TiO_2_-dye) and a counter electrode (Pt/AZO/i-ZnO/CdS/CIGS/Mo-coated glass) into a sandwich type using 60 μm thick surlyn (DuPont 1702). The working electrode was prepared by the identical method to DSSC as mentioned above. Also, the counter electrode was fabricated similar to that of single junction CIGS solar cell except for adding the Pt layer on top of the AZO film.

### Characterization

Structural characterization of the films was performed using a scanning electron microscope (SEM, Nova, Nano200) with an acceleration voltage of 10 kV, transmission electron microscopy (TEM, FEI Inc., Tecnai G2), and X-ray diffraction (XRD, Shimadzu, XRD-6000) with Cu-Kα radiation (λ = 0.15406 nm). The optical properties were measured by ultraviolet-visible spectroscopy (UV-Vis, Varian, Cary 5000). Composition analysis was carried out by an electron probe microanalyzer (EPMA, Jeol, JXA-8500 F) and energy-dispersive X-ray spectroscopy (EDX, E2V Tech. Inc.). Depth profiling was conducted by Auger electron spectroscopy (AES, Ulvac-Phi). Device performances were characterized using a class AAA solar simulator (Wacom, WXS-155S-L2) and an incident photon-to-current conversion efficiency (IPCE) measurement unit (PV measurement Inc.).

## Author Contributions

B.K.M. and D.L. planned the project. S.H.M. and S.J.P. managed and performed most detailed experiment, and S.H.K., M.W.L., J.H., J.Y.K., H.K. and Y.J.H. helped data analysis and manuscript preparation. All the authors discussed the results and commented on the manuscript.

## Supplementary Material

Supplementary Information Supplementary Information
