# Supplementary material for: Monolithic DSSC/CIGS tandem solar cell fabricated by a solution process
Source: Sci Rep. 2015 Mar 11;5:8970. doi: 10.1038/srep08970 (PMC4355678; doi:10.1038/srep08970)
Supplement: Supplementary Information [file srep08970-s1.doc]

**Supplementary Information**

Monolithic DSSC/CIGS tandem solar cell fabricated by a solution process

Sung Hwan Moon*^1^, Se Jin Park*^1,2^, Sang Hoon Kim^3^, Min Woo Lee^4^, Jisu Han^4,5^, Jin Young Kim^4^, Honggon Kim^4^, Yun Jeong Hwang^1^, Doh-Kwon Lee^4^ & Byoung Koun Min^1,5^

^1^ Clean Energy Research Center, Korea Institute of Science and Technology, Hwarang-ro 14-gil 5, Seongbuk-gu, Seoul, 136-791, Republic of Korea.

^2^ Department of Chemical and Biological Engineering, Korea University, 145, Anam-ro, Seongbuk-gu, Seoul 136-713, Republic of Korea

^3^ Center for Materials Architecturing, Korea Institute of Science and Technology, Hwarang-ro 14-gil 5, Seongbuk-gu, Seoul, 136-791, Republic of Korea.

^4^ Photo-electronic Hybrids Research Center, Korea Institute of Science and Technology, Hwarang-ro 14-gil 5, Seongbuk-gu, Seoul, 136-791, Republic of Korea.

^5^ Green School, Korea University, 145, Anam-ro, Seongbuk-gu, Seoul 136-713, Republic of Korea

^*^ These authors contributed equally to this work.

Corresponding authors: bkmin@kist.re.kr, dklee@kist.re.kr


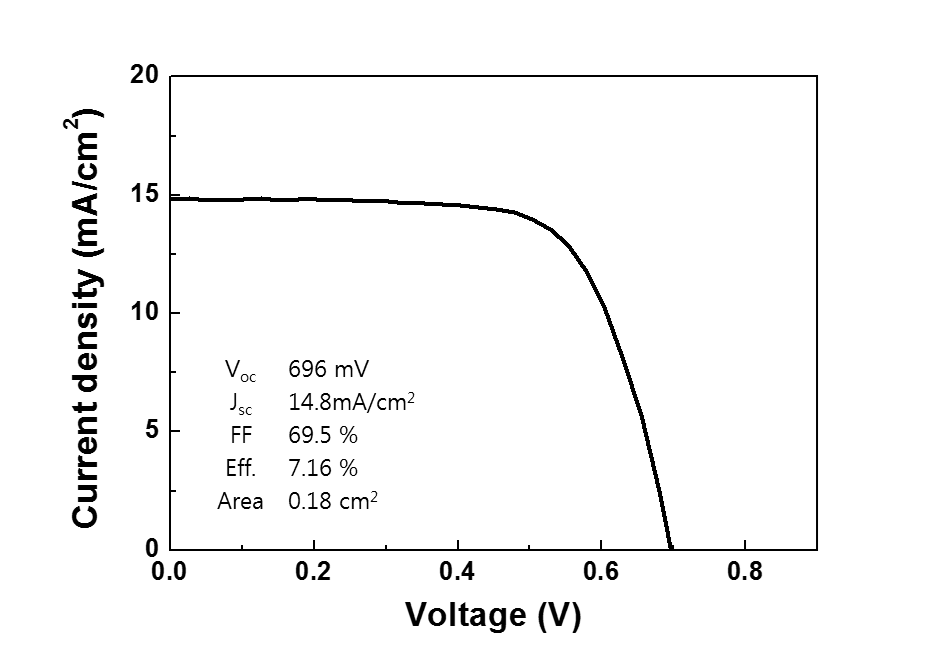


**Figure S1** The current density-voltage (*J-V)* characteristics of DSSC with the Pt layer deposited by thermal sintering method


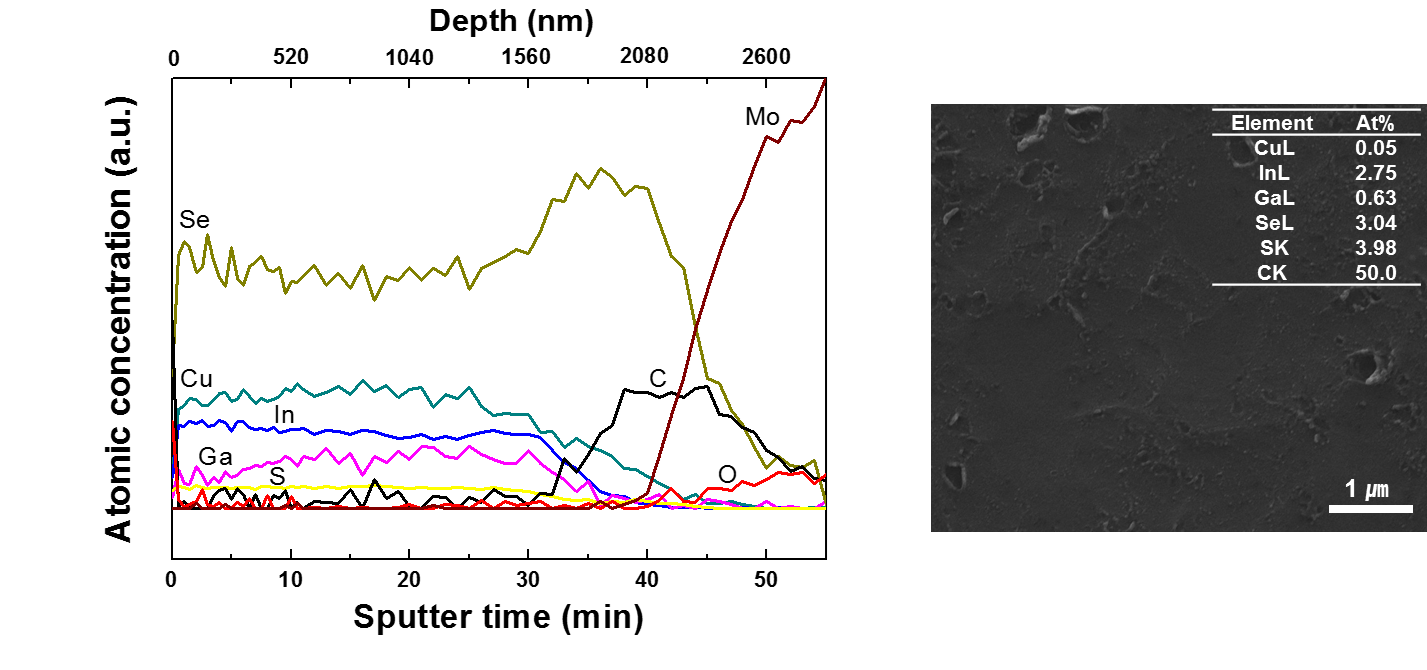


**Figure S2** The composition profile of CIGS thin film by Auger depth profiling and top-view image of SEM and EDX analysis of carbon layer which was existed between CIGS and Mo layer

**Figure S3** (a) Typical *J*−*V* curves of DSSC, CIGS, and DSSC-CIGS tandem solar cells in the dark and (b) d*V*/d*J* against 1/(*J* − *G*_sh_*V*) plots where *G*_sh_ is shunt conductance and *R*_s_ is extracted from the *y*-intercept.

**Figure S4** Reproduction of Figure 4 with a *J*–*V* curve of the simulated CIGS bottom cell included. The simulated CIGS bottom cell has *V*_oc_ = 0.445 V, *J*_sc_ = 14.6 mA/cm^2^, and FF = 0.79.

**Figure S5** Stability test of the DSSC/CIGS tandem solar cell device under standard irradiation conditions (AM 1.5G, 100 mW/cm^2^).
